# SAlign–a structure aware method for global PPI network alignment

**DOI:** 10.1186/s12859-020-03827-5

**Published:** 2020-11-04

**Authors:** Umair Ayub, Imran Haider, Hammad Naveed

**Affiliations:** 1 Department of Computing, National University of Computer and Emerging Sciences, Islamabad, 40100 Pakistan; 2 Computational Biology Research Lab, Islamabad, 40100 Pakistan

**Keywords:** Protein–protein interaction, Global network alignment, Sequence similarity, Structure similarity, Monte–Carlo algorithm

## Abstract

**Background:**

High throughput experiments have generated a significantly large amount of protein interaction data, which is being used to study protein networks. Studying complete protein networks can reveal more insight about healthy/disease states than studying proteins in isolation. Similarly, a comparative study of protein–protein interaction (PPI) networks of different species reveals important insights which may help in disease analysis and drug design. The study of PPI network alignment can also helps in understanding the different biological systems of different species. It can also be used in transfer of knowledge across different species. Different aligners have been introduced in the last decade but developing an accurate and scalable global alignment algorithm that can ensures the biological significance alignment is still challenging.

**Results:**

This paper presents a novel global pairwise network alignment algorithm, SAlign, which uses topological and biological information in the alignment process. The proposed algorithm incorporates sequence and structural information for computing biological scores, whereas previous algorithms only use sequence information. The alignment based on the proposed technique shows that the combined effect of structure and sequence results in significantly better pairwise alignments. We have compared SAlign with state-of-art algorithms on the basis of semantic similarity of alignment and the number of aligned nodes on multiple PPI network pairs. The results of SAlign on the network pairs which have high percentage of proteins with available structure are 3–63% semantically better than all existing techniques. Furthermore, it also aligns 5–14% more nodes of these network pairs as compared to existing aligners. The results of SAlign on other PPI network pairs are comparable or better than all existing techniques. We also introduce $\hbox {SAlign}^{\mathrm{mc}}$, a Monte Carlo based alignment algorithm, that produces multiple network alignments with similar semantic similarity. This helps the user to pick biologically meaningful alignments.

**Conclusion:**

The proposed algorithm has the ability to find the alignments that are more biologically significant/relevant as compared to the alignments of existing aligners. Furthermore, the proposed method is able to generate alternate alignments that help in studying different genes/proteins of the specie.

## Background

Proteins are large biomolecules that perform their functions by interacting with other biomolecules. We can represent the proteins of a particular specie as a network, where nodes in the network represent the proteins and edges show the interactions between these proteins. The amount of protein interaction data has increased significantly in recent years due to the advancement in high throughput experiments. PPI networks of two species can be compared to detect evolutionary conserved interactions. This comparison highlights the structurally and functionally conserved parts of the two networks. It can also be helpful in finding unidentified interactions [1, 2] and in drug design [3, 4]. Hence, it is crucial that the methods used by researchers to align PPI networks are precise and accurate.

The term pairwise network alignment is used for the comparison of two PPI networks. The mapping of a smaller network over the portion of a larger network is known as an alignment. There are two types of network alignments - (i) Local Network Alignment and (ii) Global Network Alignment. Local aligners use many-many mapping between the nodes [5, 6]. A single node of network A can align with multiple nodes of network B and vice versa. Local aligners can generate multiple sub-alignments. In contrast to local aligners, global aligners use one-one mapping between nodes. A single node of network A can align to a single node of network B. The primary goal of such global aligners is to match the maximum number of functionally similar nodes [1, 7–9].

Existing studies use network topology and/or sequence information to align the PPI networks. Different types of measures are used to calculate the topology. For example, HubAlign use minimum degree heuristic for calculating topology [1]. ModuleAlign develop a novel method for using topological information that is based on hierarchical clustering [7]. IBNAL develop a clique based index to measure the topology of the proteins [10]. NETAL and PROPER use local topological measures to calculate the topology [8, 9]. Similarly, previous studies use different types of heuristics to align the network. For example, HubAlign, NETAL and IBNAL use different forms of greedy algorithm for alignment. ModuleAlign uses the Hungarian algorithm while PROPER uses percolation-graph-matching algorithm for alignment. MAGNA uses genetic algorithm for alignment [11]. UAlign thoroughly investigates the different aligners and combined them to align the network pairs. UAlign conclude that the use topology does not result in high semantic similarity while prioritizing biological information may result in high semantic similarity [12]. Table 1 shows the comparison of different studies on the basis of features, topological measures, alignment heuristics, datasets, advantages and limitations.

**Table 1:** The comparison between the existing studies is presented

Method	Features	Topological method	Alignment heuristic	Datasets	Advantages	Limitations
HubAlign	Sequence + topology	Min. degree heuristic	Greedy algorithm	IntAct	Scalable better alignment in terms of no. of aligned nodes	AFS is not better as HubAlign prioritises topology
ModuleAlign	Sequence + topology + clustering based scores	Min. degree heuristic + cluster similarity scores	Hungarian algorithm	HINT	Module based (clustering) scoring matrix helps in producing quality alignment	Complexity is high
PROPER	Sequence + topology	Local network topology	Percolation graph matching algorithm	IntAct	Takes less resources and time	Align few no. of nodes
IBNAL	Functional similarity + topology	Clique-degree signature similarity	Greedy Algorithm (based on clique size)	IsoBase	Uses less resources	Go-annotations are required in alignment phase
MAGNA	Sequence only	–	Genetic algorithm	BioGRID	Efficient for alignments that required high topological quality	1-optimize the results w.r.t topology only that results in low semantic similarity 2-exponential complexity time
NETAL	Topology only	Local topological measure with iterative updates	Greedy-algorithm	IntAct	High speed	Performance is measured using topological measures only
UAlign	Sequence + topology	UAlign unifies the alignments of eight aligners which include Natalie, SPINAL, PISwap, MAGNA, HubAlign, L-GRAAL, OptNetAlign and ModuleAlign. The best features of all aligners are used to optimize the alignment w.r.t different measures				

The features used by the existing aligners, topological measures, alignment heuristics, datasets, advantages and limitations are compared

Several studies have achieved significant success in the field of global PPI network alignment. However, new methods are required to generate alignments with better semantic similarity. Moreover, the results of some of the existing studies (IBNAL and PROPER) are inefficient in terms of the number of aligned nodes.

Existing studies measure the performance of the global aligners on the basis of semantic similarity and number of align nodes. Semantic similarity is used to compare the genes/proteins based on their context. In PPI context, the semantic similarity between the proteins can be measured by calculating the similarity between the functions of the proteins instead of their sequence or structure. It is also important to note that most previous studies have used topology as pseudo measure to calculate functional/semantic similarity. A previous study has noted that topological similarity does not guarantee functional similarity and that functional similarity is best measured using a semantic similarity measure [12]. Similarly most of the previous studies have not tested their methods robustness by testing on multiple datasets. Datasets have different compilation strategies, bias and completeness level. BioGrid is a public database that archives and disseminates genetic and protein interaction data collected from over 70,000+ publications in the primary literature [13]. HINT is a public curated compilation of high-quality protein-protein interactions from 8 resources (BioGRID, MINT, iRefWeb, DIP, IntAct, HPRD, MIPS and the PDB). Interactions are filtered to remove erroneous and low-quality interactions [14].

Ideally, an aligner should align maximum number of nodes while making sure that the aligned nodes are semantically related and be tested across different datasets. This paper presents a novel method, SAlign, which in contrast to existing aligners, uses structure and sequence information to calculate biological scores instead of only sequence information. SAlign also uses the topological information of the network. The results of SAlign are compared with several existing aligners on multiple PPI networks based on the percentage of total nodes aligned and the semantic similarity of the aligned nodes. For the network pairs with high percentage of proteins with experimentally resolved 3D structures, SAlign on average achieves 3–63% higher semantic similarity than existing aligners. Moreover, it aligns 5–14% more nodes than existing aligners.

All the existing aligners and SAlign are deterministic in nature and always produce the same alignment for multiple runs. There are several cases where alternate options with very similar functional similarity are available that might be biologically more relevant. To address this issue, we presents a variant of SAlign, $\hbox {SAlign}^{\mathrm{mc}}$, that is based on Monte Carlo (MC) algorithm. $\hbox {SAlign}^{\mathrm{mc}}$ has the ability to generate multiple global alignments of the two networks with similar average semantic similarity by aligning the networks on the basis of probabilities (generated by MC) instead of the highest alignment scores.

## Results

The results of SAlign and its variant, $\hbox {SAlign}^{\mathrm{mc}}$, are compared with prominent existing aligners on BioGRID (three network pairs) and HINT (five network pairs) datasets. Existing prominent techniques include HubAlign [1], ModuleAlign [7], NETAL [8], PROPER [9], IBNAL [10] and Magna++ [15]. The performance of IsoRank [16], PISwap [17], GHOST [18], PINALOG [19], L-GRALL [20], Great [21] and SPINAL [22] have been shown to be lower than most of the above mentioned algorithms, so we did not include these algorithms in our analysis. The results of all the aligners including SAlign are validated by calculating AFS using Wang method [23]. There are two main types of validation methods – the first type is of Information Content (IC) based methods like Lin [24], Resnick [25] and Schlicker [26]. The second type is of graph based methods which include GOGO [27] and Wang [23]. IC based validation gives the semantic similarity between two nodes by counting the number of children and/or distance between the term and the closet common ancestor of both terms. IC based methods are dependent on the annotation database which is biased towards the proteins or genes which are more studied by the researchers [23]. The graph based methods use only the graph of Gene Ontology (GO). Wang is provided by several online tools (GoSemSim [28], G-SESAME [29] and NaviGo [30], *etc.*). The results of the tools vary due to the implementation differences and due to the usage of different versions of the GO database. GoSemSim is used by most recent studies for semantic similarity calculation as it uses the latest version of GO database [31–33]. Therefore, we also use GoSemSim for semantic similarity calculation.

### The results of SAlign and $\hbox {SAlign}^{\mathrm{mc}}$ on mouse-human, human-yeast and mouse-yeast pairs

As we integrate the structural information of the proteins in our methodology, we divided our dataset into 2 parts: i) species for which significant number of proteins have resolved 3D structures ii) species for which 3D structure information of enough proteins is not available. The results of the pairs which have high percentage of proteins with experimentally resolved 3D structures (Mouse-Human, Human-Yeast and Mouse-Yeast pairs) are analyzed in this section. We first compare SAlign with other aligners on the HINT database, which contains high quality PPI interactions from 8 different databases. The results of SAlign on the basis of average percentage of aligned nodes and average AFS w.r.t MF and BP are better than all existing aligners (Table 2a). For MF, the AFS of SAlign is 48–63% higher than ModuleAlign, IBNAL, NETAL and Magna++ aligners. Moreover, it aligns 7–14% more nodes than these aligners. Similarly, for BP, the AFS of SAlign is 40–52% higher and it aligns 5–10% more nodes than ModuleAlign, IBNAL, NETAL and Magna++ aligners. When we compare SAlign with PROPER, we observe that SAlign performs better albeit moderately in terms of AFS w.r.t. to BP and MF (3% and 8% respectively). However, it significantly outperforms PROPER in terms of number of nodes aligned (13% and 14% respectively for BP and MF). Furthermore, HubAlign’s performance is close to SAlign in terms of number of align nodes, but SAlign outperforms HubAlign in terms of AFS with a significant margin (13% w.r.t BP and 14% w.r.t MF).

$\hbox {SAlign}^{\mathrm{mc}}$, a variant of SAlign with the ability to generate several global alignments with similar semantic similarity shows perfomance similar to SAlign in terms of average percentage of aligned nodes and average AFS w.r.t MF and BP. The average standard deviation for $\hbox {SAlign}^{\mathrm{mc}}$ is found to be $\approx 5e^{-5}$ and $\approx 2e^{-5}$ for HINT and BioGRID datasets, respectively.

**Table 2:** Comparison between the results of SAlign (SA) and existing techniques on network pairs which have high percentage of proteins with experimentally resolved 3D structures on the basis of AFS and percentage of aligned nodes w.r.t MF and BP

Pairs	Evaluation criteria	SA	$\hbox {SA}^{\mathrm{mc}}$	HA	MA	IBN	NET	M++	PRO
*Results on HINT datasets (a)*									
Mouse human	AFS$_{MF}$	*0.58*	0.55	0.48	0.42	0.35	0.33	0.36	*0.58*
	AFS$_{BP}$	0.43	0.41	0.34	0.30	0.26	0.24	0.26	*0.45*
	Nodes$_{MF}$	*82*	*82*	78	74	72	73	76	*82*
	Nodes$_{BP}$	85	*86*	84	81	83	82	82	84
Mouse yeast	AFS$_{MF}$	0.40*	0.39	0.36	0.31	0.29	0.31	0.29	0.36
	AFS$_{BP}$	0.27*	0.26	0.25	0.23	0.21	0.22	0.21	0.25
	Nodes$_{MF}$	72	*73*	71	71	63	64	67	53
	Nodes$_{BP}$	*92*	91	90	88	76	83	83	69
Human yeast	AFS$_{MF}$	0.48*	0.46	0.46	0.26	0.30	0.26	0.26	0.42
	AFS$_{BP}$	0.35*	0.33	0.34	0.22	0.24	0.22	0.22	0.32
	Nodes$_{MF}$	*64*	63	63	60	58	60	59	57
	Nodes$_{BP}$	*76*	*76*	*76*	72	70	72	70	68
Avg.	AFS$_{MF}$	*0.49*	0.47	0.43	0.33	0.31	0.30	0.31	0.45
	AFS$_{BP}$	*0.35*	0.33	0.31	0.25	0.24	0.23	0.24	0.34
	Nodes$_{MF}$	*73*	*73*	71	68	64	66	67	64
	Nodes$_{BP}$	*84*	*84*	83	80	76	79	78	74
*Results on BioGRID datasets (b)*									
Mouse human	AFS$_{MF}$	*0.64*	0.63	0.57	0.46	0.35	0.33	0.36	0.63
	AFS$_{BP}$	*0.48*	0.47	0.43	0.35	0.27	0.26	0.28	*0.48*
	Nodes$_{MF}$	*89*	*89*	88	85	80	83	86	83
	Nodes$_{BP}$	*96*	*96*	95	93	90	93	*96*	93
Mouse yeast	AFS$_{MF}$	*0.47*	0.46	0.44	0.37	0.28	0.27	0.33	*0.47*
	AFS$_{BP}$	*0.32*	*0.32*	0.29	0.27	0.22	0.22	0.22	*0.32*
	Nodes$_{MF}$	*82*	*82*	80	80	76	77	80	58
	Nodes$_{BP}$	96	96	94	*97*	88	92	*97*	67
Human yeast	AFS$_{MF}$	0.53*	0.52	0.48	0.42	0.34	0.28	0.29	0.49
	AFS$_{BP}$	0.39*	0.38	0.35	0.33	0.27	0.23	0.24	0.38
	Nodes$_{MF}$	*74*	*74*	73	*74*	63	72	70	67
	Nodes$_{BP}$	*91*	*91*	*91*	*91*	77	*91*	90	82
Avg.	AFS$_{MF}$	*0.55*	0.54	0.50	0.42	0.32	0.29	0.33	0.53
	AFS$_{BP}$	*0.40*	0.39	0.36	0.32	0.25	0.24	0.25	0.39
	Nodes$_{MF}$	*82*	*82*	80	80	73	77	79	69
	Nodes$_{BP}$	*94*	*94*	93	*94*	85	92	*94*	79

The particular results of the best aligners are differentiated from other aligners by italic text

For HINT datasets, the average AFS of SAlign w.r.t. MF and BP is 0.49 and 0.35 respectively

SAlign on average aligns 73% and 84% node in MF and BP respectively. SAlign outperforms all other aligners on the given evaluation criteria. For BioGRID datasets, the average AFS w.r.t. MF and BP is 0.53 and 0.38 respectively. SAlign on average aligns 81% and 94% node in MF and BP respectively. ‘*’ shows that the results are statistically significant

Table 2b presents the results of different aligners on BioGRID datasets. BioGRID contains relatively dense networks as compared to HINT as it contains all interactions reported in literature. In contrast, HINT contains only high quality, manually reviewed interactions. Therefore, the noise level in BioGrid is relatively high. The AFS of SAlign is 31–89% and 25–67% higher than ModuleAlign, IBNAL, NETAL, and Magna++ aligners w.r.t MF and BP, respectively. SAlign outperforms HubAlign with 10–11% margin in terms of MF and BP. The performance of SAlign is similar or slightly higher than existing aligners in terms of number of aligned nodes except PROPER and IBNAL. SAlign aligns 11–12% higher number of nodes as compared to IBNAL. When we compare SAlign with PROPER, we observe that SAlign performs better albeit moderately in terms of AFS w.r.t. to BP and MF (2% and 4% respectively). However, it significantly outperforms PROPER in terms of number of aligned nodes (18% and 19% w.r.t MF and BP, respectively).

For global network alignment, the number of aligned nodes are as important as biological similarity (AFS). PROPER aligned lower number of nodes as compared to all existing algorithms and SAlign. We have compared the results of PROPER and SAlign for equal number of nodes as PROPER shows similar AFS for lower number of aligned nodes. For Hint datasets, for equal number of aligned nodes, the margin between the performance of SAlign and PROPER has been increased to 21% and 22% from 3 and 8% w.r.t BP and MF, respectively. Similarly, for BioGRID datasets, the margin has been increased to 7% and 11% from 2 and 4% w.r.t BP and MF, respectively. These results show that the small alignments result in high AFS as the alignment of a smaller portion of a network is easier than the complete alignment. The detailed comparison between PROPER and SAlign for equal number of aligned nodes is given in Additional file 1: section 3.

**Fig. 1:**
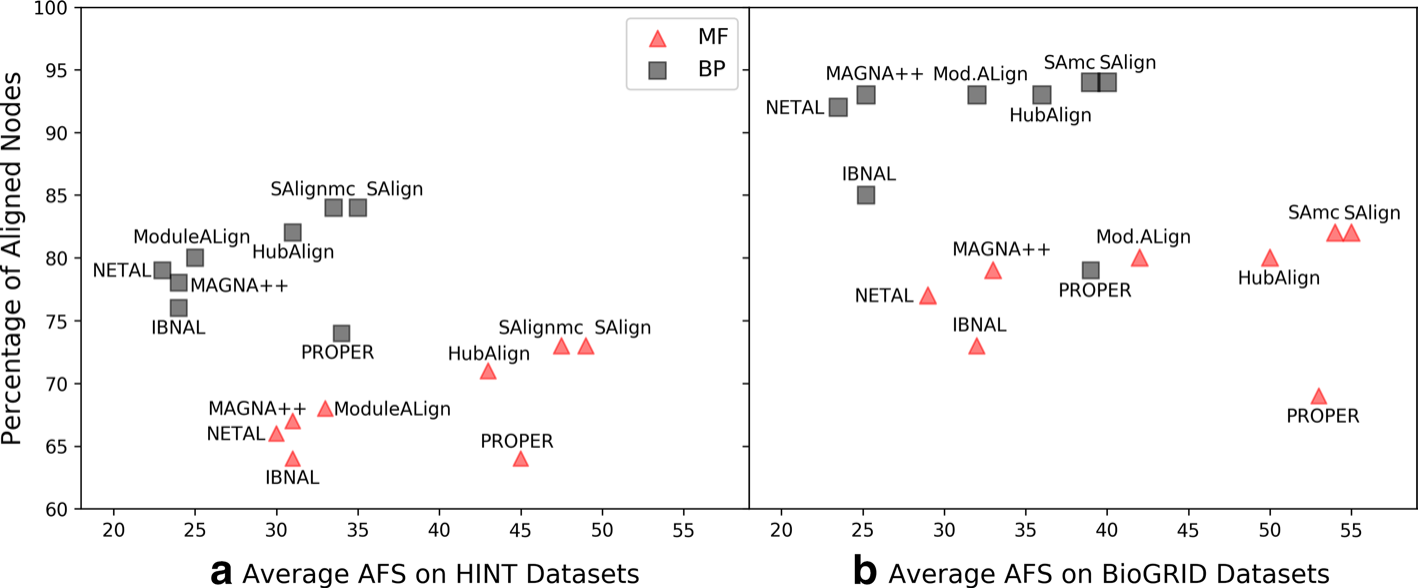
Results of all the aligners in terms of align nodes and AFS. The results of SAlign, $\hbox {SAlign}^{\mathrm{mc}}$ and existing aligners on the basis of average AFS and average percentage of aligned nodes are presented. These results are the averages of Mouse-Human, Human-Yeast and Mouse-Yeast pairs collected from HINT **a** and BioGRID **b** databases. x-axis represents the average AFS score while y-axis represents the percentage of aligned nodes

Figure 1a represents the 2D position or performance of each aligner in terms of average percentage of aligned nodes and average AFS on HINT datasets. The graphical results are shown in the form of multi-objective functions. Aligner that reaches the upper right portion is desired as this portion indicates that the aligner aligns the maximum number of biologically relevant nodes. The position of SAlign and its variant, $\hbox {SAlign}^{\mathrm{mc}}$, clearly highlight the effectiveness of the proposed technique. The performance of ModuleAlign, IBNAL, NETAL and Magna++ is inferior than PROPER, SAlign and HubAlign in both objectives (number of aligned nodes and AFS) as shown in Fig. 1a. HubAlign is inferior than SAlign and $\hbox {SAlign}^{\mathrm{mc}}$. PROPER performs relatively better as compared to previous aligners in terms of AFS but its position along y-axis is not comparable to HubAlign and SAlign. Figure 1b represents the 2D position of each aligner in terms of average percentage of aligned nodes and average AFS on the datasets collected from BioGRID. Figure 1b depicts the similar trend to Fig. 1a except for PROPER and ModuleAlign. The performance of ModuleAlign is relatively better on BioGRID datasets. PROPER outperforms all other aligners in terms of average AFS but it is inferior among all aligners in terms of number of aligned nodes. The position of SAlgin is better than all aligners for both objectives on BioGRID datasets as well.

### Comparison of SA with existing aligners on network pairs which have low percentage of proteins with experimentally resolved 3D structures

Table 3 represents the comparison of the proposed technique and existing aligners on the network pairs (HINT dataset) which have a low percentage of proteins with experimentally resolved 3D structures. The AFS of SAlign is 36–71% and 34–62% higher than ModuleAlign, IBNAL, NETAL and Magna++ aligners w.r.t MF and BP, respectively. Moreover, it also aligns 7–25% and 7–14% more number of nodes as compared to these aligners w.r.t MF and BP, respectively. SAlign outperforms HubAlign with 14–15% margin in terms of AFS and it aligns 4–7% more number of nodes as compared to HubAlign. When we compare the results of PROPER with SAlign, we observe that PROPER produces similar results to SAlign in terms of average AFS. However, SAlign outperforms PROPER with significantly high margin in terms of number of aligned nodes (15% and 17% w.r.t BP and MF, respectively). The results of $\hbox {SAlign}^{\mathrm{mc}}$ are similar to SAlign on the basis of average percentage of aligned nodes and average AFS w.r.t MF and BP. The average standard deviation for $\hbox {SAling}^{\mathrm{mc}}$ is found to be $\approx 4e^{-5}$ for Mouse-Fly and Mouse-Worm pairs.

The average results of SAlign and PROPER in terms of AFS are similar, but SAlign significantly outperforms PROPER in terms of number of aligned nodes. We compare the results of both aligners for equal number of aligned nodes. The results of SAlign are 9% and 13% higher than PROPER w.r.t MF and BP, respectively. PROPER aligns few number of nodes to produce high AFS.

**Table 3:** Comparison between the results of SAlign (SA) and existing techniques on network pairs which have low percentage of proteins with experimentally resolved structures on the basis of average AFS and average percentage of aligned nodes w.r.t MF and BP

Pairs	Evaluation criteria	Alignment algorithms							
		SA	$\hbox {SA}^{\mathrm{mc}}$	HA	MA	IBN	NET	M++	PRO
Mouse fly	AFS$_{MF}$	0.50	0.49	0.42	0.36	0.33	0.32	0.37	0.55*
	AFS$_{BP}$	0.37	0.36	0.31	0.28	0.24	0.23	0.28	0.40*
	Nodes$_{MF}$	*73*	72	67	66	58	57	63	61
	Nodes$_{BP}$	*80*	*80*	76	74	58	60	62	56
Mouse worm	AFS$_{MF}$	0.56*	0.54	0.49	0.41	0.30	0.29	0.31	0.52
	AFS$_{BP}$	0.41*	0.40	0.37	0.30	0.25	0.24	0.25	0.39
	Nodes$_{MF}$	*76*	74	73	71	62	62	64	64
	Nodes$_{BP}$	70	68	67	66	70	72	*76*	73
Avg.	AFS$_{MF}$	*0.53*	0.52	0.46	0.39	0.32	0.31	0.34	*0.53*
	AFS$_{BP}$	0.39	0.38	0.34	0.29	0.25	0.24	0.27	*0.40*
	Nodes$_{MF}$	*74*	73	70	69	60	59	64	63
	Nodes$_{BP}$	*75*	74	72	70	66	66	69	65

The particular results of the best aligners are differentiated from other aligners by italic text

SAlign performs well in terms of average AFS w.r.t MF and BP and it also outperforms existing aligners in terms of average percentage of align nodes. ‘*’ shows that the results are statistically significant

**Fig. 2:**
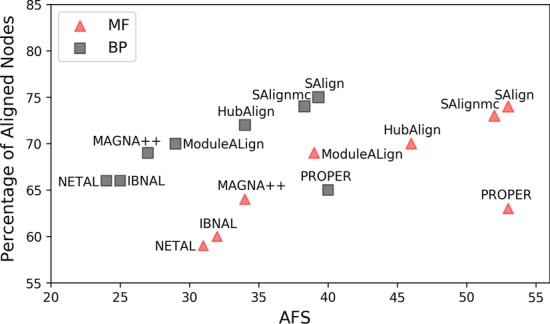
Results of all the aligners for Mouse-Worm and Mouse-Fly pairs. The results of SAlign, $\hbox {SAlign}^{\mathrm{mc}}$ and existing aligners on the basis of average AFS and average percentage of aligned nodes are presented. These results are the averages of Mouse-Worm and Mouse-Fly pairs collected from HINT. x-axis represents the average AFS score while y-axis represents the average percentage of aligned nodes

Figure 2 represents the 2D position or performance of each aligner in terms of average percentage of aligned nodes and average AFS for the datasets that have low percentage of proteins with 3D structures. The position of SAlign and its variant, $\hbox {SAlign}^{\mathrm{mc}}$ is significantly better than other aligners w.r.t both axis except PROPER. The position of PROPER and SAlign along x-axis is close but the position of PROPER along y-axis is not comparable to SAlign. The relative position of SAlign and its variant, $\hbox {SAlign}^{\mathrm{mc}}$, is better than all existing aligners despite of low percentage of structure availability for worm and fly networks.

### Monte Carlo based alignments

Although the results of SAlign, based on greedy alignment algorithm, are similar to $\hbox {SAlign}^{\mathrm{mc}}$, it has the limitation of fixed alignment for every run. Sometimes, subsets of the alignments generated by the existing aligners, including SAlign, are not biologically meaningful. $\hbox {SAlign}^{\mathrm{mc}}$, a variant of SAlign, can handle this situation as it incorporates MC based selection to select a protein out of the *N* most suitable proteins from network 2 to align to a protein in network 1. It can generate alternate alignments with similar average AFS. Their is no existing study which supports such feature.

As $\hbox {SAlign}^{\mathrm{mc}}$ has the ability to generate multiple alignments for different runs, it can be used in studying the networks of the species that are not well-studied. For most of the species, the PPI data is not complete and contains noisy interactions. In this particular case, alternate alignments are useful as one can pick the most biologically meaningful alignment. Alternate alignments can help in studying different interactions between the groups of proteins (of two different species).

For example, if we run the basic SAlign several times on Mouse-Human pair, it always aligns *P*09450 gene with *Q*13451 gene. The MF and BP scores for this pair are 0.35 and 0.29, respectively. Conversely, $\hbox {SAlign}^{\mathrm{mc}}$ aligns *P*09450 gene with different genes on every run. From the ten different alignments, we pick three genes (*Q*13485, *Q*96*EC*8 and *P*84077) which are aligned by $\hbox {SAlign}^{\mathrm{mc}}$ with *P*09450 gene. The MF scores of these three genes are 0.71, 0.66 and 0.39, respectively. The BP scores of these genes are 0.51, 0.48 and 0.29, respectively. This shows that $\hbox {SAlign}^{\mathrm{mc}}$ has the ability to align the more biologically similar genes.

### Optimization of $\alpha$ and $\beta$

**Fig. 3:**
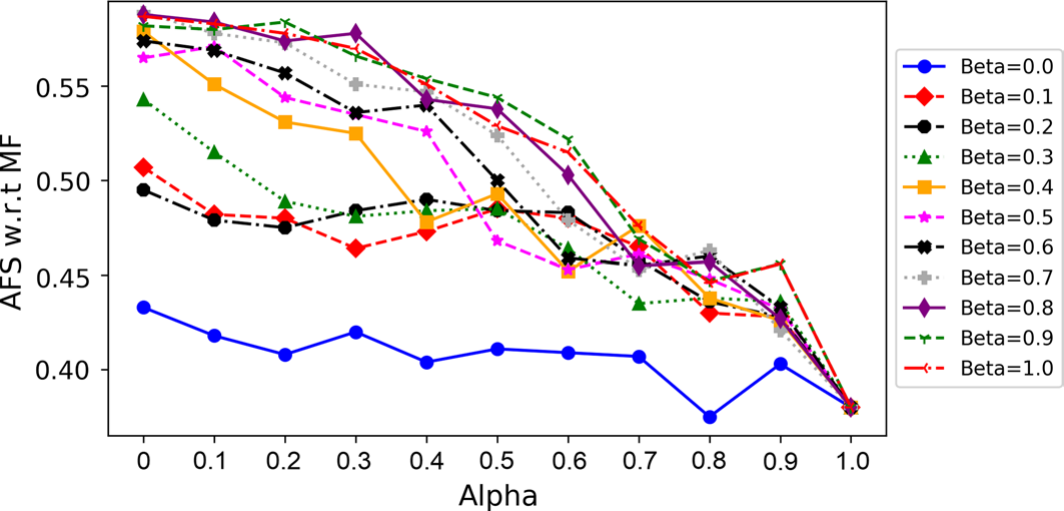
$\alpha /\beta$ Tuning for Mouse-Human Pair. The AFS w.r.t MF on Mouse-Human pair is shown. AFS is scaled along y-axis while $\alpha$ is scaled along x-axis. Every line represents the MF scores for each value of $\alpha$ at some specific $\beta$. High value of $\alpha$ indicates the high contribution of topological measure while high values of $\beta$ indicates the high contribution of sequence similarity

To maximize the semantic similarity score of PPI networks in terms of BP and MF, the values of $\alpha$ and $\beta$ are tuned using grid search. $\alpha$ is used to assign the weights to topological and biological similarity scores. $\beta$ is used to assign the weights to sequence and structure similarity scores while computing biological scores. Figure 3 shows the results of SAlign on different values of $\alpha$ and $\beta$ for Mouse-Human pair. The values of $\alpha$ and $\beta$ have a similar impact on MF and BP scores. SAlign achieves the best performance for the species pairs that have high percentage of proteins with available 3D structures when the values of $\alpha$ and $\beta$ are set to 0.1 and 0.7, respectively. For the species pairs that have low percentage of proteins with available 3D structure, SAlign best performs when the values of $\alpha$ and $\beta$ are set to 0.1 and 0.9, respectively. We have tuned the values of $\alpha$ and $\beta$ on Mouse-Human, Mouse-Yeast and Yeast-Human pairs that are collected from HINT database. BioGRID datasets are tested on these values to test the generality of SAlign. The Mouse-Worm and Mouse-Fly pairs tuned and produce better results on different set of values as worm and fly contain only 2% and 3% proteins that have available 3D structures. All the results of $\hbox {SAlign}^{\mathrm{mc}}$ are computed using the same values of $\alpha$ and $\beta$.

### Species-wise tuning of $\alpha$ and $\beta$

The effect of $\alpha$ and $\beta$ is analyzed on all species independently. Every pair is tested on all combinations of $\alpha$ and $\beta$. The best and worst performance for each specie is reported in Table 4. Max and min represents the maximum and minimum AFS score of each specie pair, respectively, on some specific values of $\alpha$ and $\beta$. Average-i presents the average results for the first three pairs (Mouse-Human, Mouse-Yeast, and Yeast-Human), while Average-ii presents the average results of Mouse-Worm and Mouse-Fly pairs. Generally, the maximum values in terms of AFS are achieved when the biological information is higher than topological information. The maximum performance of SAlign has been recorded when the sequential information is 70–90% for different species pairs. The minimum values in terms of AFS are achieved when the topological portion is higher than the biological portion in the final alignment score. This analysis highlighted that the biological relevance is not associated with topological measures, instead it is highly correlated with biological information (sequence and structure). Our results are consistent with the results of UAlign in terms of using topology to measure biological similarity [12].

**Table 4:** The Max–Min performance of SAlign, on HINT datasets, achieved on the basis of AFS w.r.t BP and MF

Species pairs	MF: Max	MF: Min	BP: Max	BP: Min
Mouse-human	0.59 (0.0,0.7)	0.38 (0.8,0.0)	0.45 (0.0,0.8)	0.27 (1.0,0.0)
Yeast-human	0.43 (0.0,0.8)	0.29 (0.9,0.0)	0.30 (0.0,0.9)	0.21 (0.9,0.0)
Mouse-yeast	0.49 (0.2,0.9)	0.27 (1.0,0.0)	0.36 (0.0,1.0)	0.22 (1.0,0.0)
Mouse-worm	0.57 (0.2,1.0)	0.35 (0.4,0.0)	0.42 (0.2,0.9)	0.25 (0.9,0)
Mouse-fly	0.52 (0.0,0.9)	0.32 (0.8,0.0)	0.39 (0.0,1.0)	0.24 (0.8,0.0)
Average-i	0.50	0.33	0.37	0.23
Average-ii	0.54	0.33	0.40	0.24

A combination of two values in parenthesis represents the combination of $\alpha$ and $\beta$ for which the max-min performance is achieved

After analyzing the results of SAlign on different set of values of $\alpha$ and $\beta$, we concluded that to achieve best performance of SAlign, the value of $\alpha$ should lie between 0.1 and 0.2 while the value of $\beta$ should be in range of 0.7 to 0.9. As the percentage of available 3D structures increases, the value of $\beta$ should be decreased.

## Discussion

This study presents a novel approach to align the two PPI networks by integrating topological, sequential and structural information. Combining the results from three specie pairs that have sufficient percentage of 3D resolved structures available, we show that the average AFS is increased by 8–63% and 3–52% in terms of MF and BP respectively. The average percentage of aligned nodes is increased by 7–14% and 5–13% in terms of MF and BP respectively.

The global alignment problem can be considered as a multi-objective problem. Ideally, the aligners should align the maximum possible number of nodes with high semantic similarity. The general trend among existing aligners is that they either perform better in terms of AFS or percentage of aligned nodes. For example, the results of PROPER on the basis of AFS w.r.t MF and BP are better than all techniques excluding SAlign for all pairs. However, PROPER aligns much fewer percentage of nodes. From Table 2b, we can see that the results of PROPER on the basis of average AFS are better than all existing aligners excluding SAlign and $\hbox {SAlign}^{\mathrm{mc}}$ but it aligns 6–17% and 8–19% fewer nodes than other aligners w.r.t MF and BP, respectively. The results of ModuleAlign, NETAL and Magna++ are relatively higher than existing aligners in terms of average percentage of align nodes but these aligners do not perform well in terms of AFS. On average, the performance of IBNAL is inferior among all aligners in terms of average AFS and percentage of align nodes. In contrast to the results of existing aligners, SAlign and $\hbox {SAlign}^{\mathrm{mc}}$ produce accurate results in terms of AFS as well as percentage of aligned nodes. The model with few numbers of aligned nodes might fail to capture all the pathways or fail to capture the complete pathways. So, the global aligner that produces high number of nodes is better in terms of completeness/correctness as compared to the model that aligns a smaller number of nodes. The graphical representation of the above analysis is given by Figs. 1 and 2.

$\hbox {SAlign}^{\mathrm{mc}}$ has the advantage of generating the several global alignments with similar AFS. This is advantageous as sometimes subsets of the alignments generated for a pair of PPI networks are not biologically meaningful, therefore generating alternate alignments can help in achieving biological meaningful network alignments. Moreover, alternate alignments can help in studying the interactions of the proteins of the species that are not well-studied in the literature.

One of the key hypothesis supported by UAlign was that the topological information does not guarantee biological relevance. Different studies (ModuleAlign, IBNAL, NETAL and Magnaa++) used different types of topological methods to align the networks, but these aligners did not perform better in terms of semantic similarity (Tables 2 and 3). We have tuned the topological, sequential and structural weights using grid search and observed that the highest semantic similarity has been achieved when the biological information was high. The lowest semantic similarity has been achieved when the topological information was high (Table 4). Furthermore, the behaviour depicted by Fig. 3 support the above analysis. As the topological information is getting high (towards right side of x-axis), the height of all the curves is falling down. All the lines decreased irrespective of $\beta$ value.

We also noted that the AFS w.r.t MF is higher than the AFS w.r.t BP. This general trend among the values of MF and BP is due to the purity of their GO-terms. The GO-terms of the molecular functions are specific and well-defined (precise semantics). When we compare the GO-terms of the biological processes, the difference is high as biological process are large processes and they involve multiple molecular functions. The GO-terms of biological processes are mostly generic and less-pure. The number of aligned nodes are higher in case of BP as compared to MF due to the same reason as mentioned above. The GO-terms of MF are specific and less annotated (functions of the proteins are not completely known). In contrast, the generic activity of the proteins is mostly known (e.g. proteins are involve in metabolism process).

## Conclusion

In this paper, we have proposed a novel method to align two PPI networks. Existing studies used topological and/or sequence information to align the networks. This paper presented a novel approach that integrated structure, sequence and topological information. As the structural information can help inferring function better than sequence information therefore the inclusion of structural information results in more biologically relevant alignments. We have compared the results of the proposed approach with multiple prominent tools and found that our approach is significantly better than existing studies on majority of the PPI network pairs. The performance of SAlign in terms of average AFS is higher than the existing aligners (8–63% and 3–52% w.r.t MF and BP, respectively) for the specie pairs that have high percentage of proteins with experimentally resolved 3D structures. It also aligns higher number of nodes than other aligners (7–14% and 5–13% w.r.t MF and BP, respectively) for above mentioned specie pairs. $\hbox {SAlign}^{\mathrm{mc}}$ incorporates MC based selection to generate alternate alignments with similar average AFS.

## Methods

### Overview

Biological networks contain some proteins that are more important than others in terms of their topology or biological function. Proteins usually interact with many other proteins. On the basis of these interactions, nodes of the network can be divided into several types. The first type are bottleneck nodes (proteins/nodes with high betweenness centrality (measure of centrality of a node in a graph/network)), which have a low degree (number of direct connection of a node with its neighbours) but connect two clusters of nodes together [1, 34]. Removal of the bottleneck nodes causes distortion in the network and might split a network into multiple sub-networks. Biologically, these nodes can be essential for proper functioning of a pathway. The second type of nodes are hub nodes, which have a higher degree. These nodes are more conserved and their mutation rate is slow as compared to the normal nodes [1, 35]. The third type are peripheral nodes, which are less important and have a low degree. Removal of these nodes usually does not disturb the topology of a network.

**Fig. 4:**
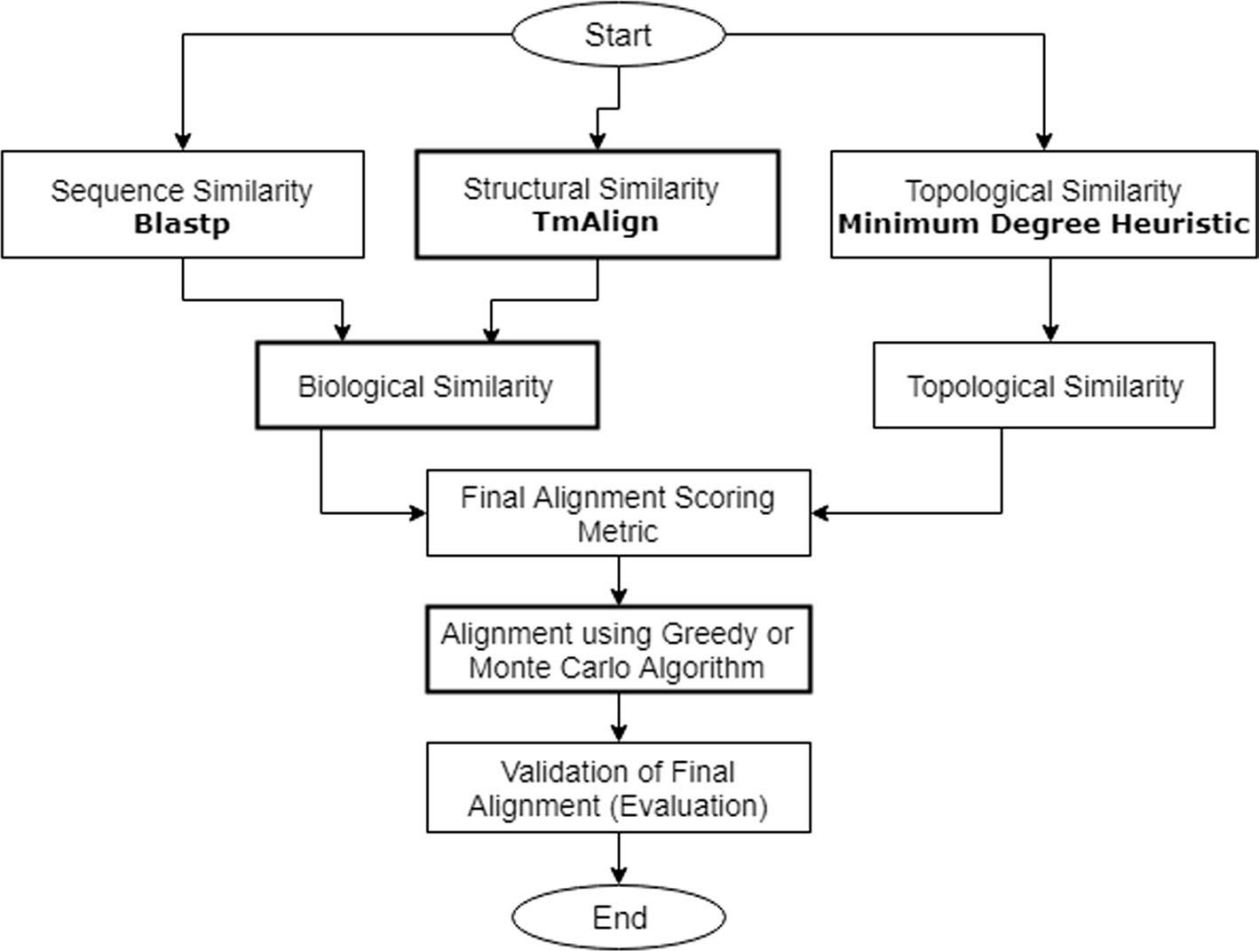
The flowchart of SAlign. Major contribution of the study includes the integration of structural information in the alignment process. A variant of SAlign, $\hbox {SAlign}^{\mathrm{mc}}$, is introduced which includes Monte Carlo based alignment algorithm

The alignment process starts by computing topological and biological scores. Following HubAlign, SAlign computes the topological score using recursive minimum degree heuristic algorithm, while the biological score is computed using protein sequence (amino acid) and structure (experimentally resolved 3D structure) similarity matrices. The calculated biological and topological matrices are combined to produce the final alignment scoring matrix. Every node of the first network is compared with all the nodes of the second network and the best match in terms of alignment score is selected. $\hbox {SAlign}^{\mathrm{mc}}$, aligns the nodes on the basis of probabilities generated from alignment scores through MC instead of picking the highest-ranked pair. After alignment, SAlign uses Wang method [23] to find the semantic similarity of the aligned nodes. Nodes which are similar in terms of biological process, molecular function and topology should have high semantic similarity value. We also report the percentage of total nodes aligned after the alignment process is complete. A flowchart of the proposed technique is shown in Fig. 4 and the pseudo-code of the proposed methodology is given in Additional file 1: Algorithm 1.

### Topological scoring matrix

Topological score (TS), which represents the importance of a particular node in a network, can be computed in two different ways: locally – by just counting the degree of the node and globally – by finding the importance of all the nodes with which that particular node is directly or indirectly connected. The global method ensures that bottleneck and hub nodes get higher weights than other nodes.

Minimum degree heuristic is a global method used for measuring topology. Minimum degree heuristic deletes nodes with the lowest degree first, and then progressively deletes nodes of higher degrees. The algorithm keeps removing the nodes until the degree reaches set threshold *d*. Initially, all the nodes’ and edges’ weights are initialized to 0 and 1, respectively. The algorithm updates the weights using equations from Additional file 1: Equation 1 to Equation 6. The details of minimum degree heuristic method can be seen in [1].

### Biological scoring matrix

The biological matrix in our methodology is an aggregate of sequence and structure similarity matrices as shown in Eq. 1.

1 $$\begin{aligned} B_{i,j} = (1 - \beta ) \times SS_{i,j} + \beta \times SQ_{i,j} \end{aligned}$$

where *i* and *j* are the nodes of network *G*1 and network *G*2, respectively. $SS_{i,j}$ and $SQ_{i,j}$ represent the structure similarity score and sequence similarity score of the nodes of the two networks. $B_{i,j}$ represents the biological similarity score of nodes *i* and *j*. The parameter $\beta$, is used to assign weightage to structure and sequence while computing biological scores. If $\beta$ is set to 0.7 it means that the sequence gets $70\%$ weightage while the structure gets $30\%$.

To calculate the sequence similarity matrix, every node, *u*, of network *G*1 is compared with every node, *v*, of network *G*2 using the BLAST_p method [36]. To calculate the structure similarity matrix, we have aligned all the available protein structures of network *G*1 with all the available protein structures of network *G*2 using TM-Align [37].

### Final alignment score calculation

The topological and biological scores are combined to calculate the final alignment score (Eq. 2).

2 $$\begin{aligned} A_{i,j} = \alpha \times T_{i,j} + (1 - \alpha ) \times B_{i,j} \end{aligned}$$

where *i* and *j* are the nodes of network *G*1 and network *G*2, respectively. $T_{i,j}$ represents the topological similarity score between the nodes *i* and *j*. $B_{i,j}$ represents the biological similarity scores between nodes *i* and *j*. $A_{i,j}$ represents the final alignment score assigned to node pairs. $\alpha$ is the trade-off constant between topological and biological similarity score. $\alpha$ ranges between 0 and 1. If the value of $\alpha$ is set to 0.1, it indicates that the biological score has $90\%$ contribution in the final alignment score.

### PPI network alignment

#### Greedy based alignment algorithm

Once the alignment score is computed for every pair of nodes of the two networks, the greedy algorithm is applied for network alignment. For every node *u* of network *G*1, all the nodes of network *G*2 are compared and the best matching node pairs in terms of alignment score are selected. Each node of network *G*2 can be aligned with the node of network *G*1 only once. Neighbors of the aligned nodes are prioritized during the alignment process. In this way, the algorithm maintains topological consistency within the alignment. This procedure continues until all or maximum number of nodes of a small network are aligned.

#### Monte Carlo based alignment algorithm

One of the big limitations of the greedy algorithm is the fixed alignment. To cater to this limitation and generate multiple different alignments, we have designed a semi_greedy alignment algorithm based on MC. The greedy algorithm always picks the best matching node but MC based algorithm picks the node from top *n* nodes. The alignment scores of the top *n* nodes are normalized using Eq. 3. These normalized scores are used by MC (Eq. 4) for generating the selection probabilities of top *n* nodes. The final node selection is based on these probabilities. All experiments are performed using $n=10$. The pseudo code of the proposed work is given in Algorithm 1.

3 $$\begin{aligned} NS_i = n_i / s \quad \forall \, n_i \in Tn \end{aligned}$$

where, *s* is the summation of the alignment scores of top *n* nodes while *Tn* is the list of top *n* nodes. $NS_i$ is the normalized score.

4 $$\begin{aligned} Prob_i = \frac{exp(-(best-NS_i)/KT)}{\sum _{i=1}^{n} exp(-(best-NS_i)/KT)} \end{aligned}$$

where, *best* is the node with maximum normalized alignment score and used as a reference for the MC model. *K*, and *T* are the constant used by MC algorithm. The product of $K \dot{T}$ is set to 0.1. $NS_i$ is the normalized score of the $i^{th}$ node from the list of top *n* nodes.

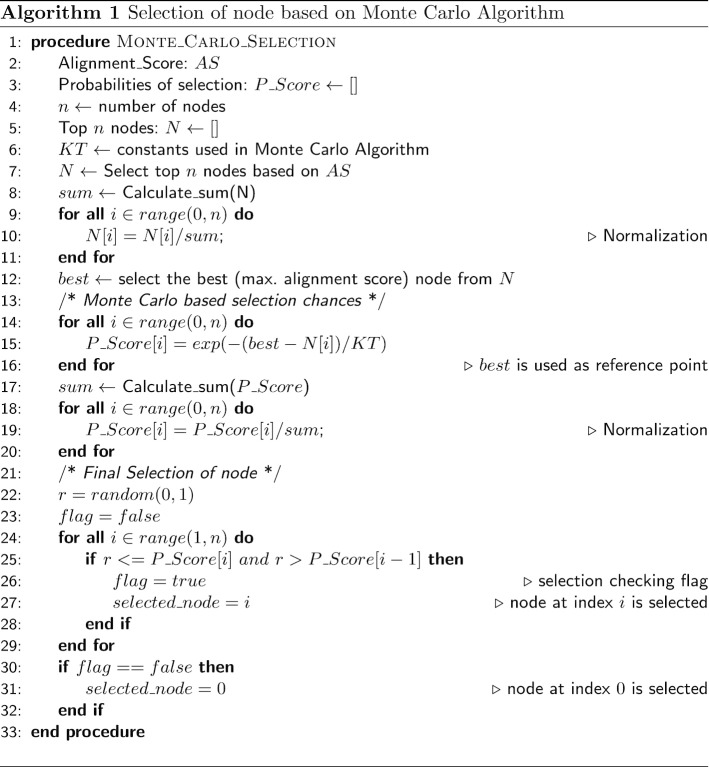



### Evaluation of alignment

We have evaluated the final alignment on the basis of percentage of aligned nodes and Average Functional Similarity (AFS) which is further categorized into Molecular Function (MF) and Biological Process (BP). Molecular activity performed by the proteins is known as MF. The large biological processes in which proteins are involved is referred to as BP. AFS is computed in two stages. In the first stage, GO (gene ontologies) terms are extracted and the similarity between the extracted GO terms is calculated in the second stage. Different methods like average, max or Best-Matched-Average (BMA) can be used to combine multiple GO-terms of a single protein. We have used BMA method to combine multiple GO-terms. The semantic similarity w.r.t MF and BP is calculated using a graph based method, Wang. The detailed working of Wang method can be seen in [23]. AFS is calculated using Eq. 5.

5 $$\begin{aligned} AFS_{c} = \frac{1}{|V_{1}|} \times \sum _{u \epsilon V_{1}} s_{c}(u, g(u)) \end{aligned}$$

where $s_{c}$ is the semantic similarity of nodes *u* and *g*(*u*), calculated by Wang, for type *c* ($c \epsilon BP or MF$). $|V_{1}|$ is the length of the alignment (number of pairs). The average of semantic similarities of the complete alignment (pairs of aligned proteins) is referred to as AFS.

**Table 5:** Data statistics: number of nodes, edges, and percentage of proteins with 3D resolved structure is presented

	HINT					BioGRID		
Species	Mouse	Human	Yeast	Worm	Fly	Mouse	Human	Yeast
Nodes	744	10791	5036	4486	7498	1584	8932	4036
Edges	1229	47427	19085	11496	25679	4574	125765	63161
Structure %	17	43	29	2	3	24	53	38

### Dataset

We have tested our proposed method on HINT (5 network pairs) and BioGrid (3 network pairs) datasets. The details of each network is given in Table 5. The first row of the table has the species names. Second and third rows have the number of nodes and edges, respectively. The fourth row represents the percentage of proteins with experimentally resolved 3D structures.

## Supplementary information

**Additional file 1.** The topological measure used by SAlign. The comparison between SAlign and PROPER when equal number of aligned nodes are considered.

## Data Availability

SAlign and SAlign^mc^ algorithms and datasets are available at GitHub: https://github.com/cbrl-nuces/SAlign. All the datasets we used in this paper are also publicly available (cited in the manuscript).

## Abbreviations

PPI: Protein–protein interaction
BLAST: Basic local alignment search tool
TM-Align: Template modeling based alignment
BioGRID: Biological general repository for interaction datasets
HINT: High-quality interactomes
AFS: Average functional similarity
MF: Molecular function
BP: Biological process
MC: Monte Carlo
