# Supplementary material for: SAlign–a structure aware method for global PPI network alignment
Source: BMC Bioinformatics. 2020 Nov 4;21:500. doi: 10.1186/s12859-020-03827-5 (PMC7640460; doi:10.1186/s12859-020-03827-5)
Supplement: Supplementary file 1 — Additional file 1. The topological measure used by SAlign. The comparison between SAlign and PROPER when equal number of aligned nodes are considered. [file 12859_2020_3827_MOESM1_ESM.pdf]

SUPPORTING INFORMATION

SAlign – A Structure Aware Method for Global PPI Network Alignment

Umair Ayub, Imran Haider, and Hammad Naveed*

Computational Biology Research Lab, Department of Computer Science, National University of Computer and Emerging Sciences, Islamabad, 44000, Pakistan

E-mail: hammad.naveed@nu.edu.pk

1. Topological Scoring Matrix

Topological score (TS) represents the importance of a particular node of the network which can be computed in two different ways. Locally – by just counting the degree of the node and globally – by finding the importance of all the nodes to which that particular node is directly or indirectly connected. In case of local method, the bottleneck nodes get lower weights as these nodes have low degree which may result in splitting of network into many disconnected sub-networks. So we have used the global method (minimum degree heuristic algorithm), which ensures that the bottleneck and hubs get higher weights as compared to other nodes. Equation 3 is used to compute TS. We compute the topological score following HubAlign[1]. Briefly, minimum degree heuristic algorithm deletes the nodes which have degree less than some threshold (set manually). In the initialization step, all nodes' weights are initialized with 0 and all the edges' weights are initialized with 1 using Equation 1 and Equation 2.

$$w(e) = \begin{cases} 1 & e \,\epsilon\, E \\ 0 & otherwise \end{cases} \tag{1}$$

Where $w(e)$ is the weight of edge e. If e belongs to super set E then the weight e is set to 1, otherwise 0.

$$w(u) = 0 \;\; \forall \; u \,\epsilon\, V \tag{2}$$

Where $w(u)$ is the weight of node u. All the node weights are initialized with zero to avoid any bias towards the nodes that are assigned high values during initialization. After initialization step, the weights are adjusted using Equation 3.

$$
\begin{cases}
\forall v \epsilon N(u) : w(v) = w(v) + w(u) + w(u,v) & deg(u) = 1 \\
\forall v_1, v_2 \epsilon N(u) : w(v_1, v_2) = w(v_1, v_2) \\
+ \frac{w(u) + \sum_{v \epsilon N(u)} w(u,v)}{\frac{|N(u)||N(u)-1)}{2}} & deg(u) > 1
\end{cases}
\tag{3}
$$

Where u and v represent the nodes of two different networks while $w(u)$ and $w(v)$ represent the weights of nodes u and v, respectively. deg represents the degree of any particular node. $N(u)$ represents the neighbors of node u.

The minimum degree heuristic algorithm removes the nodes which have lowest degree first, and then progressively removes the nodes of higher degree as shown in Equation 3. The algorithm keeps removing the nodes until the degree reaches the threshold d. The importance score is assigned to each node using Equation 4.

$$
S(v) = w(v) + \lambda \sum_{u \epsilon V} w(u,v) \tag{4}
$$

The importance score $S(v)$, calculated in Equation 4, depends on already calculated node and edge weights. The value of λ is set to 0.1 following[1]. The edge weights are added to the weight of node v to get the final importance score $S(v)$.

$$
S(v) = \frac{S(v)}{max_{v \epsilon V} \ S(v)} \tag{5}
$$

Where $S(v)$ represents the node's importance score corresponding to its own network. To generate the topological similarity matrix, each node of network $G1$ is compared with every node of network $G2$ using Equation 6.

$$
TS(u,v) = min(S(u), S(v)) \tag{6}
$$

The minimum node weight from both of the nodes' weights is assigned to pair (u, v).

2. Pseudocode of the Proposed Technique

Algorithm 1 SAlign: The Proposed Approach to Align the Two PPI Networks

```
procedure SALIGN
    Net1 = Network1 and Net2 = Network2
    Sequence_metrix: SQ ← []
    Structure_metrix: SS ← []
    Degree Threshold: d ← 10
    for all v ∈ V do
        W(v) ← 0
        E(v1, v2) ← 1
    end for
    for all v ∈ V do
        if degree(v) < d then
            Calculate node and edges weights using Equation 1 and Equation 2
        end if
    end for
    for all v ∈ V do
        Calculate node importance score using Equation 3
    end for
    for all v ∈ Net1, u ∈ Net2 do
        Calculate topological scoring matrix using Equation 6
    end for
    for all u ∈ Net1, v ∈ Net2 do
        SQ(u, v) = BLAST(u, v)
        SS(u, v) = TM_Score(u, v)
    end for
    Calculate biological scoring matrix using SQ and SS matrices
    for all u ∈ Net1, v ∈ Net2 do
        Calculate final alignment score matrix
    end for
    for all u ∈ Net1 do
        for all v ∈ Net2 do
            Select un-visited node, v for which node u has the maximum Alignment Score
            Update the weights of neighbors of nodes u and v
        end for
    end for
end procedure
```

3. Comparison of SAlign and PROPER for Equal Number of Aligned Nodes

SAlign produces better results as compared to all existing aligners in terms of number of aligned nodes and AFS. The number of nodes aligned by all existing aligners are reasonable except PROPER while the AFS produce by PROPER is reasonable as compared to all existing aligners. To the best of our knowledge, SAlign is the only aligner that produce high AFS with high number of aligned nodes.

SAlign outperforms PROPER in many cases but the difference between the average performance of both aligners is not as high as for other aligners. So, we have decided to further analyze the results of PROPER and SAlign. Although the performance of SAlign is higher than PROPER in most of the cases, to gain the confidence we make comparison between these two aligners for equal number of align nodes. When we consider equal number of aligned nodes, the difference between the performance of SAlign and PROPER has been increased. On average, for equal number of aligned nodes, SAlign outperforms PROPER by 10% and 11% w.r.t MF and BP, respectively (from Table S1). This shows that the alignment of a smaller portion of a network is easier than the alignment of a complete network. Large alignments (high number of nodes) usually result in low AFS as the error of miss-aligned nodes is propagated to the end of the alignment process. Despite of this fact, SAlign manage to produce highest number of aligned nodes with maximum possible semantic similarity.

Table S2 presents the statistical comparison between the results of SAlign and PROPER for equal number of aligned nodes. For Mouse-Human pair, the results of both aligners are similar. For Muse-Fly pair, the results of PROPER are better than SAlign with a small margin. The statistical results does not prove the previous statement (the results of both aligners are statistically similar). For remaining five pairs, the results of SAlign are statistically better than PROPER with 95% confidence.

Table S1: Comparison between the results of SAlign and PROPER on the basis of AFS for equal number of aligned nodes.

Database	Pair	Evaluation w.r.t AFS	Models	
			SAlign	PROPER
HINT	Mouse-Human	MF	0.58	0.58
		BP	0.45	0.45
HINT	Mouse-Yeast	MF	**0.50**	0.36
		BP	**0.34**	0.25
HINT	Yeast-Human	MF	**0.53**	0.42
		BP	**0.39**	0.32
HINT	Mouse-Worm	MF	**0.62**	0.52
		BP	**0.47**	0.39
HINT	Mouse-Fly	MF	0.53	**0.55**
		BP	0.39	**0.40**
BioGRID	Mouse-Human	MF	**0.64**	0.63
		BP	0.48	0.48
BioGRID	Mouse-Yeast	MF	**0.59**	0.47
		BP	**0.39**	0.32
BioGRID	Yeast-Human	MF	**0.55**	0.49
		BP	**0.40**	0.38

Table S2: The statistical results of SAlign and PROPER on the basis of AFS for equal number of aligned nodes. Two sample Independent T-Test has been applied to validate the results.

Database	Pairs	P-Values (MF)	P-Values (BP)
HINT	Mouse-Human	0.96	0.57
HINT	Mouse-Yeast	$2.6e^{-15}$	$6.3e^{-10}$
HINT	Yeast-Human	$1.9e^{-42}$	$5.1e^{-16}$
HINT	Mouse-Worm	$0.13e^{-15}$	$1.4e^{-6}$
HINT	Mouse-Fly	0.82	0.61
BioGRID	Mouse-Human	0.32	0.31
BioGRID	Mouse-Yeast	$7.7e^{-28}$	$4.5e^{-12}$
BioGRID	Yeast-Human	$2.2e^{-8}$	0.65

References

(1) Hashemifar, S.; Xu, J. Hubalign: an accurate and efficient method for global alignment of protein–protein interaction networks. *Bioinformatics* **2014**, *30*, i438–i444.
